# Local fractal dimension of collagen detects increased spatial complexity in fibrosis

**DOI:** 10.1007/s00418-023-02248-8

**Published:** 2023-11-08

**Authors:** Dylan T. Casey, Karolyn G. Lahue, Vitor Mori, Jacob Herrmann, Joseph K. Hall, Béla Suki, Yvonne M. W. Janssen-Heininger, Jason H. T. Bates

**Affiliations:** 1 Department of Medicine, University of Vermont Larner College of Medicine, 149 Beaumont Ave, Burlington, VT 05405 USA; 2 Complex Systems Center, University of Vermont, Burlington, VT USA; 3 Department of Pathology and Laboratory Medicine, University of Vermont Larner College of Medicine, Burlington, VT USA; 4 Department of Biomedical Engineering, University of Iowa, Iowa City, IA USA; 5 Department of Biomedical Engineering, Boston University, Boston, MA USA

**Keywords:** Fractal, Fibrosis, Morphological analysis, Collagen, Aging, Lung

## Abstract

Increase of collagen content and reorganization characterizes fibrosis but quantifying the latter remains challenging. Spatially complex structures are often analyzed via the fractal dimension; however, established methods for calculating this quantity either provide a single dimension for an entire object or a spatially distributed dimension that only considers binary images. These neglect valuable information related to collagen density in images of fibrotic tissue. We sought to develop a fractal analysis that can be applied to 3-dimensional (3D) images of fibrotic tissue. A fractal dimension map for each image was calculated by determining a single fractal dimension for a small area surrounding each image pixel, using fiber thickness as the third dimension. We found that this local fractal dimension increased with age and with progression of fibrosis regardless of collagen content. Our new method of distributed 3D fractal analysis can thus distinguish between changes in collagen content and organization induced by fibrosis.

**Supplementary Information:**

The online version contains supplementary material available at 10.1007/s00418-023-02248-8.

## Introduction

Fractals are objects that exhibit the characteristic of self-similarity, meaning that they have a similar appearance regardless of the length scale at which they are viewed (Mandelbrot 1967). Such objects can be assigned a non-integer, or fractal, dimension, which has proven useful for analyzing and classifying images of fibrotic histopathological or computed tomography (CT) samples (Moal et al. 2002; Rodriguez et al. 1995). Histologic pulmonary interstitial pathologies such as idiopathic pulmonary fibrosis (IPF) remain challenging to characterize because of their spatial heterogeneity, unusual presentation, and a lack of methods for quantifying histologic features. This has culminated in fluctuating diagnostic standards (Raghu et al. 2018; Smith 2022).

The calculation of fractal dimension is usually performed in only two dimensions on planar binary images, which misses essential information regarding the intensities of the image pixels (Pentland 1984). This third dimension can be captured using techniques such as differential box-counting (Sarkar and Chaudhuri 1994, 1992), but this typically provides a single global fractal dimension for the entire image, thereby discounting the spatial heterogeneity of naturally occurring objects. Conversely, the locally connected fractal dimension (LCFD) provides a distribution of fractal dimensions for an image (Voss and Wyatt 1993; Landini et al. 1995; Ketipearachchi and Tatsumi 2015), but is only defined for binary (black & white) images. Multifractal analysis also discerns multiple levels of scaling in an image but cannot specify regions of high and low complexity (Posadas et al. 2001; Barreira et al. 1997; Reljin et al. 2000).

There is thus a need for a method that can be applied to the fractal analysis of histopathologic samples that characterizes both 2-dimensional spatial information in the image plane together with intensity-associated information in the third dimension. To meet this need, we extended the LCFD approach to include all pixel intensity values in an image. We refer to this new method as the local connected fractal surface dimension (LCFSD). In the present study, we apply LCFSD to the analysis of micrographs of both normal and fibrotic lung tissue stained for collagen.

## Materials and methods

### Mouse models

We obtained histologic samples and hydroxyproline content data from C57BL/6NJ mice undergoing three different protocols. These data have been previously reported by Anathy et al. (2018) in a different context and are presented herein as a reference point for multifractal analysis described below. All animal use and treatment protocols were reviewed and approved by the Institutional Animal Care and Use Committee at the University of Vermont. Pulmonary fibrosis was induced with either bleomycin (5 U kg^−1^ body weight, APP Pharmaceuticals) or recombinant adenovirus Ad-TGFβ1 (5 × 10^8^ PFU, provided by J. Gauldie, McMaster University) administered oropharyngeally, as previously described (Aesif et al. 2009; Anathy et al. 2012). The control for each method was phosphate-buffered saline (PBS) and Ad-Ctr (Vector Biolabs), a harmless form of the virus, respectively. We summarize the three experimental protocols below:

1. Bleomycin-induced fibrosis in young mice: Bleomycin (BLM) was instilled into the lungs to induce fibrosis in 8-week-old male mice with another aged-matched group receiving PBS as a control. The lungs from mice instilled with BLM were harvested on day 14 and day 28. The entire lung was fixed in 10% formalin and embedded in paraffin for histology. The collagen content of the superior lobe in each mouse of a duplicate experiment was quantified via a hydroxyproline assay. If any mice showed rapid decline in health, they were euthanized to eliminate suffering.
2. Bleomycin-induced fibrosis in aged mice: Eighteen-month-old mice were obtained from the National Institute of Aging (NIA) and instilled with BLM or PBS. The lungs from instilled mice were harvested on day 21 and only mice instilled with BLM were harvested on day 42. The left lobe was fixed in 10% formalin. The same animals were used for the histology (left lobe) and the hydroxyproline assay.
3. TGFβ1-induced fibrosis: Eight-week-old mice were instilled with either Ad-Ctr or Ad-TGFβ1. The experimental procedure was performed with two different timelines. First timeline: Lungs from both groups were harvested after 14 days and the Ad-TGFβ1 group again on day 28. Second timeline: Lungs from both groups were harvested after 21 days and the Ad-TGFβ1 group again at day 42. The entire lung from the 14-day (14d) procedure was fixed in 10% formalin and embedded in paraffin for histology. The superior lobes from the lungs in the 21-day (21d) experiment were used for the hydroxyproline assay.

### Histological quantification with Picrosirius red

This procedure was previously described by Anathy et al. (2018) and is recreated here. Paraffin-embedded whole lung samples were sliced in 5-µm sections and mounted on slides. They were stained with Picrosirius red (0.1% Sirius Red F3BA, Pfaltz & Bauer, S03695) in saturated picric acid to allow visualization of collagen content. Monochromatic images were taken using an Olympus SZX12 dissecting microscope. In order to reduce bias when selecting regions, these images were overlaid with a grid whose individual squares approximated the area of an image at ×20  magnification, and the square coordinates were randomly generated to select 10 parenchymal regions. If a selected region contained an edge, blank space, airway, or debris, it was discarded, and a new coordinate generated until a total of 10 had been selected. Images of these sections were taken using an Olympus BX50 at ×20 magnification with circularly polarized light, representing an area of 442 μm × 590 μm. This was done by aligning the analyzer (U-P115, Olympus) and polarizer (U-P110, Olympus) so that the background was as dark as possible (Whittaker et al. 1994). Once the polarizer was optimized for a particular slide, it was not changed until all 10 regions were captured.

The images were composed of 1600 × 1200 24-bit pixels with pixel edge length of 0.369 µm. For processing, the images were decomposed in MATLAB 2022a (The Mathworks Inc., Natick, MA, USA) into three 8-bit channels corresponding to hue, saturation, and intensity. Saturation values in the range 0–142 and intensity values in the range 0–15 were excluded. Additionally, hue values between 129 and 229 were disregarded because they represented neither background nor collagen (Rich and Wittaker 2005). The remaining hue values were grouped into the ranges red 0–9 and 230–255, orange 10–38, yellow 39–51, and green 52–128. Each of these ranges corresponds to the thickness of a collagen fiber, with red being the thickest and green being the thinnest. The hue values were then shifted and normalized to the highest red value within an experiment. The 10 samples for each animal were combined to give a mean collagen area fraction and standard deviation. Violin plots were generated with code from the Github repository (Bechtold 2016).

### Locally connected fractal surface dimension (LCFSD)

A fractal dimension is a systematic way of quantifying fractal structure by describing how an object changes as the length scale at which it is observed changes (Mandelbrot 1983). One commonly used fractal metric is the box-counting dimension. A square grid of box length $L$ is overlayed on an object, and the number of squares, $N\left( L \right)$, that the object occupies is determined. The fractal dimension is defined to be (Mandelbrot 1967)

1 $$D = \mathop {\lim }\limits_{L \to 0} \frac{\log N\left( L \right)}{{\log 1/L}}$$

For each pixel in an image, we take a region $R$ centered at the pixel $r$. The region includes $w$ pixels on each side of $r$. If we let $h = 2w + 1$, then $R$ is an $h \times h$ pixel square centered at $r$. $R^{\prime}$ is $R$ with all pixels that are not connected to *r* removed. $R^{\prime}$ is divided into subregions of nesting squares centered on $r$ of sizes $S_{L} = \left\{ {3, 5, \ldots ,\left( {h - 2} \right), h} \right\}$. Let $f$ be the set of pixel values for the square subregion $L \in S_{L}$ and define ${\mathcal{F}}$ to be the global maximum of all $f$ in the entire image set. We then rescale $f$ by

2 $$f^{*} = h\frac{f}{{\mathcal{F}}}$$

For each $L$ in $S_{L}$,

3 $$N\left( L \right) = \mathop \sum \limits_{x,y} \min \left( {f^{*} \left( {{\text{x}},{\text{y}}} \right), L} \right)$$

where $x$ and $y$ the coordinates of a pixel in the image plane. This expression for $N\left( L \right)$, Eq. (3), is then substituted into Eq. (1) to give the locally connected fractal surface dimension for $r$. The fractal dimension value is valid for values between 0 and 3. Code used to perform this calculation is available at https://github.com/dylantcasey/LCFSD.

### LCFSD application

**Fig. 1:**
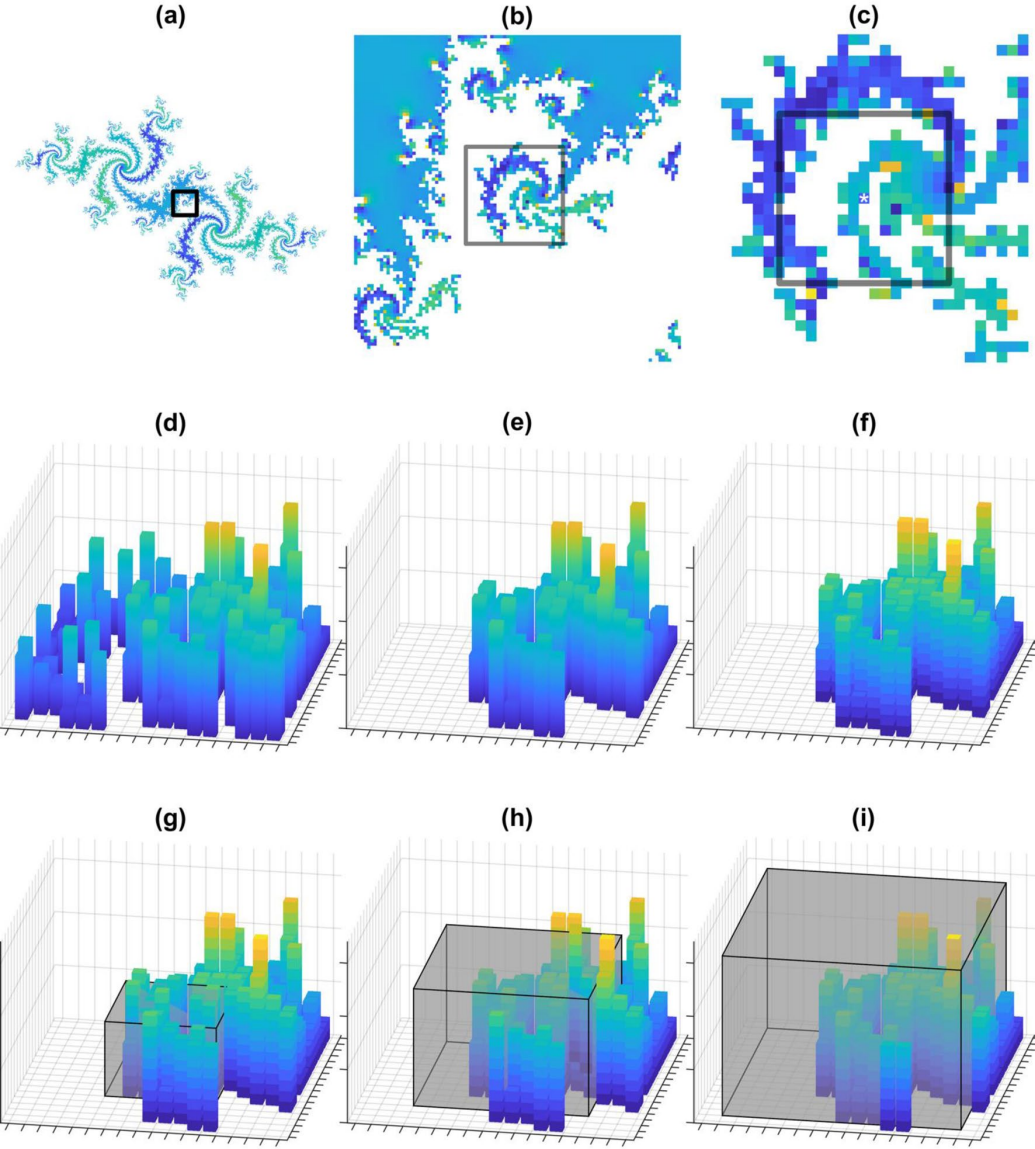
Illustration of LCFSD process. **a** Julia set for $c=-0.5285-0.5285i$, colored based on the magnitudes. **b** Zoomed-in square from (**a**). **c** Zoomed-in square from (**b**) square represents 17 × 17 area used for LCFSD analysis with an asterisk identifying the center pixel. **d** Rotated square in 3D from (**c**) where the height represented the magnitudes. **e** Elimination of unconnected regions from (**d**). **f** Discretization of (**e**). **g** Intersection of a 7 × 7 × 7 shaded box with (**f**). **h** Intersection of an 11 × 11 × 11 shaded box with (**f**). **i** Intersection of a 15 × 15 × 15 shaded box with (**f**)

Processed and normalized hue values were used for fractal analysis. Additionally, all pixels in clusters of five or fewer, as well as those less than $w=8$ pixels from an edge, were excluded from the analysis. The maximum square side length, $h$, was selected as 17-pixel widths (Supplementary Fig. 1), corresponding to 6.3 μm. Centering a $17\times 17$ square on a pixel of nonzero hue value (Fig. 1a–c), the pixels connected to the center pixel were determined and all other values excluded (Fig. 1d, e). A pixel was defined as being connected if any of its eight adjacent neighbor pixels had a nonzero hue value. The hue values that provided the third dimension of information represented as the height of each pixel were rescaled via Eq. (2) and were binned into 17 levels by rounding up to the nearest whole number to create a 17 × 17 × 17 cube of voxels (Fig. 1f). This region was divided up into cubic boxes of side lengths, $L,$ equal to 3-, 5-, 7-, 11-, 13-, 15-, and 17-pixel widths, all centered on the pixel in question, as illustrated in Fig. 1g–i. The logarithm of the number of occupied voxels within a box was plotted against the logarithm of box size ($L$) From Eq. (1), the slope of the regression line through this relationship then provided the LCFSD for the corresponding pixel. This process was repeated for each nonzero pixel in the image. A histogram of the LCFSD for each image was produced.

### Julia sets as test exemplars

**Fig. 2:**
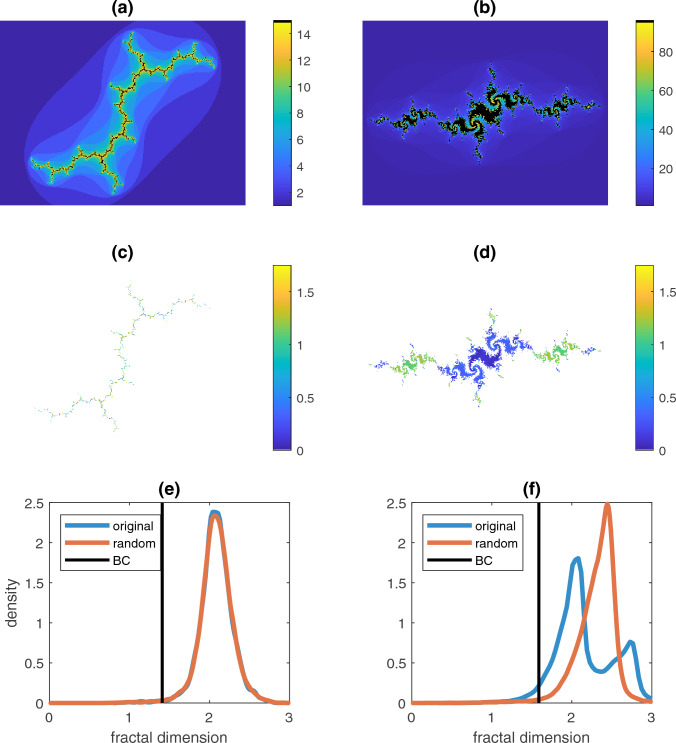
LCFSD applied to whole Julia sets. **a** Julia set for ${\varvec{c}}={\varvec{i}}$, colored by escape iteration. **b** Julia set for $c=-1.15+0.215i$, colored by escape iteration. **c** Julia set from (**a**) colored by magnitudes. **d** Julia set from (**b**) colored by magnitudes. **e** LCFSD distribution for Julia set in (**c**), including box-counting dimension in black (BC) and LCFSD of randomized magnitudes in orange. **f** LCFSD distribution for Julia set in (**d**), including box-counting dimension in black and LCFSD of randomized magnitudes in orange

We constructed images from Julia sets in order to test the performance of the LCFSD on objects having known properties. Julia sets are defined over the complex plane, $z$, by the iterative function

4 $$z_{n + 1} = z_{n}^{2} + c$$

where $\left| c \right| \le 2$ is a fixed complex parameter and $n$ is the iteration number. $z_{n + 1}$ was considered part of the Julia set if $\left| {z_{n + 1} } \right| \le 2$ (Fatou 1917; Julia 1918; Brolin 1965). For all points excluded from the Julia set, the last iteration before they left the set was taken as their escape time. We generated Julia sets using $c = - 0.5285 - 0.5285i$ with 97 iterations of Eq. (4) (Fig. 1), $c = i$ with 15 iterations of Eq. (4) (Fig. 2a, c; this is also known as the Dendrite Fractal (Carleson and Gamelin 1993)), and $c = - 1.15 + 0.215i$ with 96 iterations of Eq. (4) (Fig. 2b, d). The values of $\left| z \right|$ obtained after the final iteration of Eq. (4) in each case were taken as the third dimension for LCFSD analysis. As a negative control, images that were non-fractal in the third dimension were obtained by randomly shuffling the $z$ values within each box. These synthetic images consisted of 1386 × 1386 pixels so as to have roughly the same number of pixels as the histological images.

### Multifractal analysis

Post-processed, binarized images of the stained lung tissue samples were exported to FracLac (Karperien 2013), a plugin for ImageJ (Schindelin et al. 2012) and their multifractal spectra, $f\left( \alpha \right)$, were calculated, where $\alpha$ is the singularity strength. The grid of boxes of length $L$ used to compute the fractal dimension in each case was positioned by FracLac in order to minimize the number of grid squares containing only empty space. A digitized filled circle was analyzed as a nonfractal control. We generated a digitized Hénon map to analyze as a multifractal control (Hénon 1976). The singularity width, $\Delta \alpha = \alpha_{{{\text{max}}}} - \alpha_{{{\text{min}}}}$, was used to determine the degree of multifractality, with larger $\Delta \alpha$ meaning greater multifractality and monofractals having $\Delta \alpha = 0$(Drożdż et al. 2009). $f\left( \alpha \right)$ was then used to calculate the $\alpha$ skewness, $A_{\alpha }$, defined as

5 $$A_{\alpha } = \frac{{\Delta \alpha_{L} - \Delta \alpha_{R} }}{{\Delta \alpha_{L} + \Delta \alpha_{R} }}$$

where $\Delta \alpha_{L} = \alpha_{{0}} - \alpha_{{{\text{min}}}}$, $\Delta \alpha_{R} = \alpha_{{{\text{max}}}} - \alpha_{{0}}$, and $\alpha_{{0}}$ is the location of the maximum in $f\left( \alpha \right)$ (Drożdż and Oświȩcimka 2015). An $A_{\alpha }$ value near 0 indicates that multifractality occurs at all scales equally, whereas positive and negative values of $A_{\alpha }$ indicate that multifractality occurs at large scales and small scales, respectively.

### Statistical analysis

All statistical tests were performed with MATLAB 2022a. One-way analysis of variance (ANOVA) was used to determine differences within an experiment for hydroxyproline data and collagen pixel percent area. Post hoc multiple comparisons utilized the Tukey–Kramer method (Tukey 1949; Kramer 1956). Distributions of hue values and fractal dimension distributions were compared via a Kruskal–Wallis test (Kruskal and Wallis 1952). Every group within an experiment was sampled equally. Pairwise comparisons used the Dunn-Šidák approach when a significant difference was detected by the Kruskal–Wallis test (Dunn 1964; Šidák 1967). Statistical significance was taken as *p* < 0.05.

## Results

Figure 1 illustrates the process of calculating the LCFSD for a single pixel. Julia sets for $c=i$ and $c=-1.15+0.215i$ are illustrated in black with surroundings colored by their escape times in Fig. 2a and b, respectively. Below each are the sets colored by their magnitudes in Fig. 2c and d. The set in Fig. 2c has little variation in magnitude whereas the center of the set in Fig. 2d is flanked by satellites of noticeably higher magnitude. The Dendrite Fractal has a period-2 orbit and the second Julia set has a period-168 orbit. The LCFSD for the two sets is shown as the blue curves and their negative control is shown as the orange curves in Fig. 2e and f, respectively. The LCFSD in Fig. 2e has a unimodal distribution, which changes very little when the magnitudes of the pixels in the Julia sets are shuffled randomly to eliminate the fractal character of the third dimension (orange curve). In contrast, the LCFSD in Fig. 2e is bimodal but reverts to a unimodal distribution when the magnitudes are shuffled. This method thus successfully incorporates information not in the planar dimension and detects correlations in the third dimension.

**Fig. 3:**
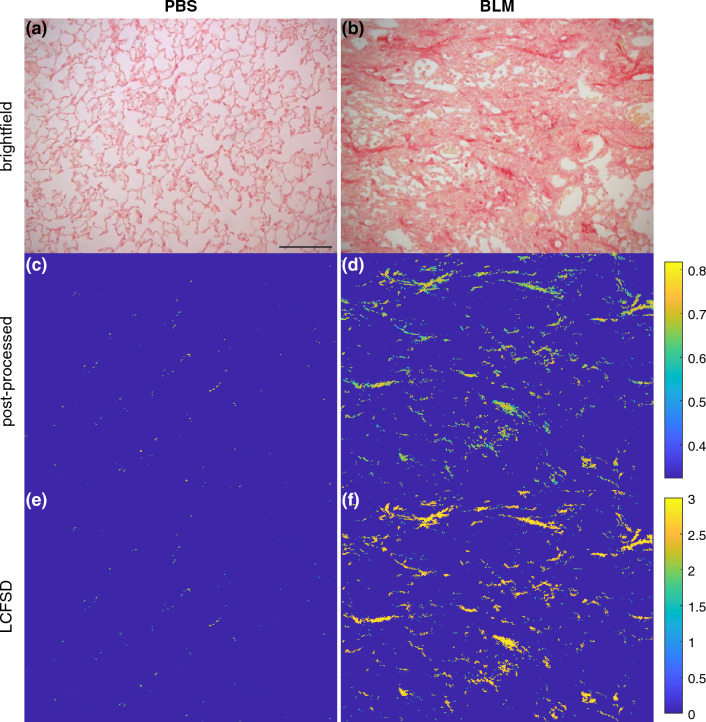
Examples of histology from Anathy et al. (2018). **a** Brightfield image of a representative PBS image from the bleomycin-induced experiment of young mice. Scale bar 100 μm. **b** Brightfield image of a representative image from the bleomycin experiment. **c** Post-processed version of (**a**) used for LCFSD calculations with a color bar shown right of (**d**) for normalized hue values. **d** Post-processed version of (**b**) used for LCFSD calculations with the same color scale as (**c**). **e** LCFSD for each pixel of (**c**) with a color bar (shown right of (**f**)) for the dimension of a pixel. **f** LCFSD for each pixel in (**d**) with the same color scale as (**e**)

Figure 3 shows representative brightfield images from Anathy et al. (2018) for a healthy lung (3a) and a bleomycin-induced fibrotic lung without treatment (3b). The roughly circular structures in the image of the healthy tissue are normal alveoli. In the fibrotic image, some alveoli structures with thickened walls remain, but most of this is replaced by solid tissue (i.e., collagen). Figure 3c and d show the post-processed versions of the corresponding brightfield images, following our preparations for LCFSD analysis. The post-processed healthy lung images are mostly empty because the magnification does not have enough light to show normal collagen levels in a tissue that is homeostatically regulated. In contrast, a large amount of collagen that exhibits birefringence reveals most of the underlying structure in the fibrotic image. The last two panels (3e, 3f) are LCFSD calculations for the post-processed images. Each pixel is colored based on its fractal dimension, illustrating locations of high and low complexity.

**Fig. 4:**
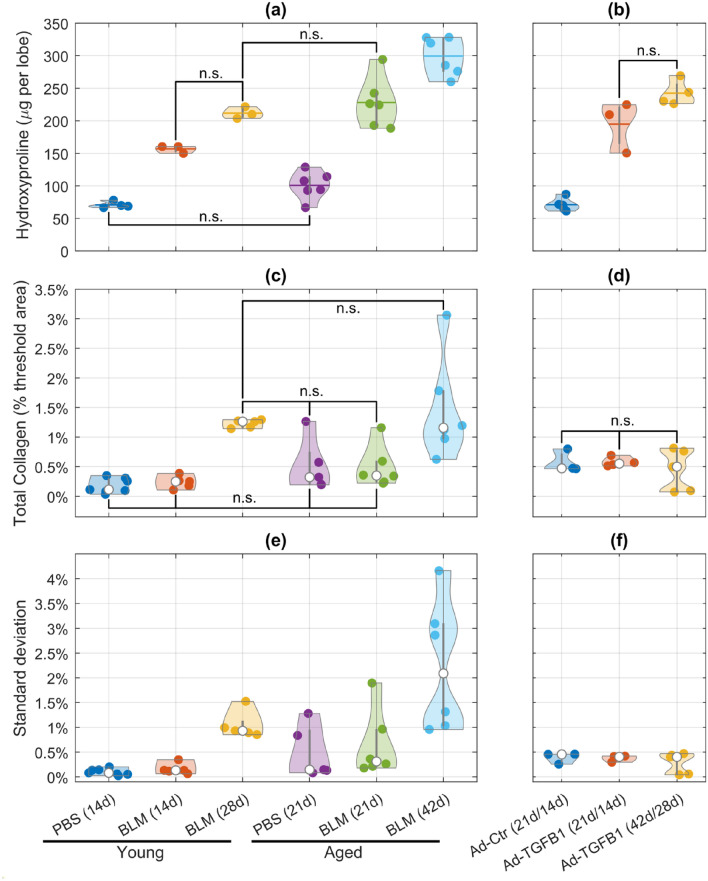
Hydroxyproline content for **a** bleomycin-induced experiment of young PBS 14d (*n* = 4), BLM 14d (*n* = 3), and BLM 28d (*n* = 3) mice and aged PBS 21d (*n* = 6), BLM 21d (*n* = 6), and BLM 42d (*n* = 6) mice; **b** TGFβ1-induced experiment with groups Ad-Ctr 21d (*n* = 5), Ad-TGFβ1 21d (*n* = 3), and Ad-TGFβ1 42d (*n* = 4) from Anathy et al. (2018). All other combinations of group comparisons within an experiment not labeled were statistically significant. Average PSR quantification for **c** bleomycin-induced experiment of young PBS 14d (*n* = 7), BLM 14d (*n* = 5), and BLM 28d (*n* = 5) mice and aged PBS 21d (*n* = 5), BLM 21d (*n* = 6), and BLM 42d (*n* = 6) mice; **d** TGFβ1-induced experiment with groups Ad-Ctr 14d (*n* = 3), Ad-TGFβ1 14d (*n* = 4), and Ad-TGFβ1 28d (*n* = 5). Average standard deviation of PSR quantification for each animal in **e** bleomycin-induced experiment of young PBS 14d (*n* = 7), BLM 14d (*n* = 5), and BLM 28d (*n* = 5) mice and aged PBS 21d (*n* = 5), BLM 21d (*n* = 6), and BLM 42d (*n* = 6) mice; **f** TGFβ1-induced experiment with groups Ad-Ctr 14d (*n* = 3), Ad-TGFβ1 14d (*n* = 4), and Ad-TGFβ1 28d (*n* = 5). Each point represents an individual animal whose 10 images have been averaged

As reported previously by Anathy et al. (2018), collagen content, as determined by the hydroxyproline assay (Woessner 1961; Kliment et al. 2011), was increased as a result of the induction of fibrosis by both bleomycin and Ad-TGFβ1. Both young and aged PBS groups showed no statistical difference in collagen content (Fig. 4a). The young BLM 28d did not test differently from the young BLM 14d and aged BLM 21d. The only other case of no difference was Ad-TGFβ1 21d compared to Ad-TGFβ1 42d (Fig. 4b). All other pairwise comparisons in all experiments were significantly different (*p* < 0.01).

In the polarized Picrosirius red (PSR) images, collagen was quantified as the ratio of the combined area of all the collagen pixels to the total tissue area per animal (Fig. 4c, d). Aged animals typically had higher median values of this ratio compared to young animals. The bleomycin-induced experiment had significant differences for the young BLM 28d compared to the other young groups, PBS 14d (*p* = 0.0040) and BLM 14d (*p* = 0.0148). The collagen area for the aged BLM 42d was different from every group except the young BLM 28d: young PBS 14d (*p* = 0.0002), young BLM 14d (*p* = 0.0010), aged PBS 21d (*p* = 0.0148), and aged BLM 21d (*p* = 0.0074). All other pairwise comparisons were not significant (Fig. 4c). All pairwise comparisons were not significantly different in the Ad-TGFβ1 experiment (Fig. 4d). The intra-animal standard deviations for the young BLM 28d and aged BLM 42d were generally much higher than the other groups (Fig. 4e, f). One animal from each of the following groups, BLM 28d, PBS 21d, and BLM 21d, also had a standard deviation in those ranges, exceeding 1%. The standard deviation for animals in the Ad-TGFβ1 experiment were low and consistent between each other.

**Fig. 5:**
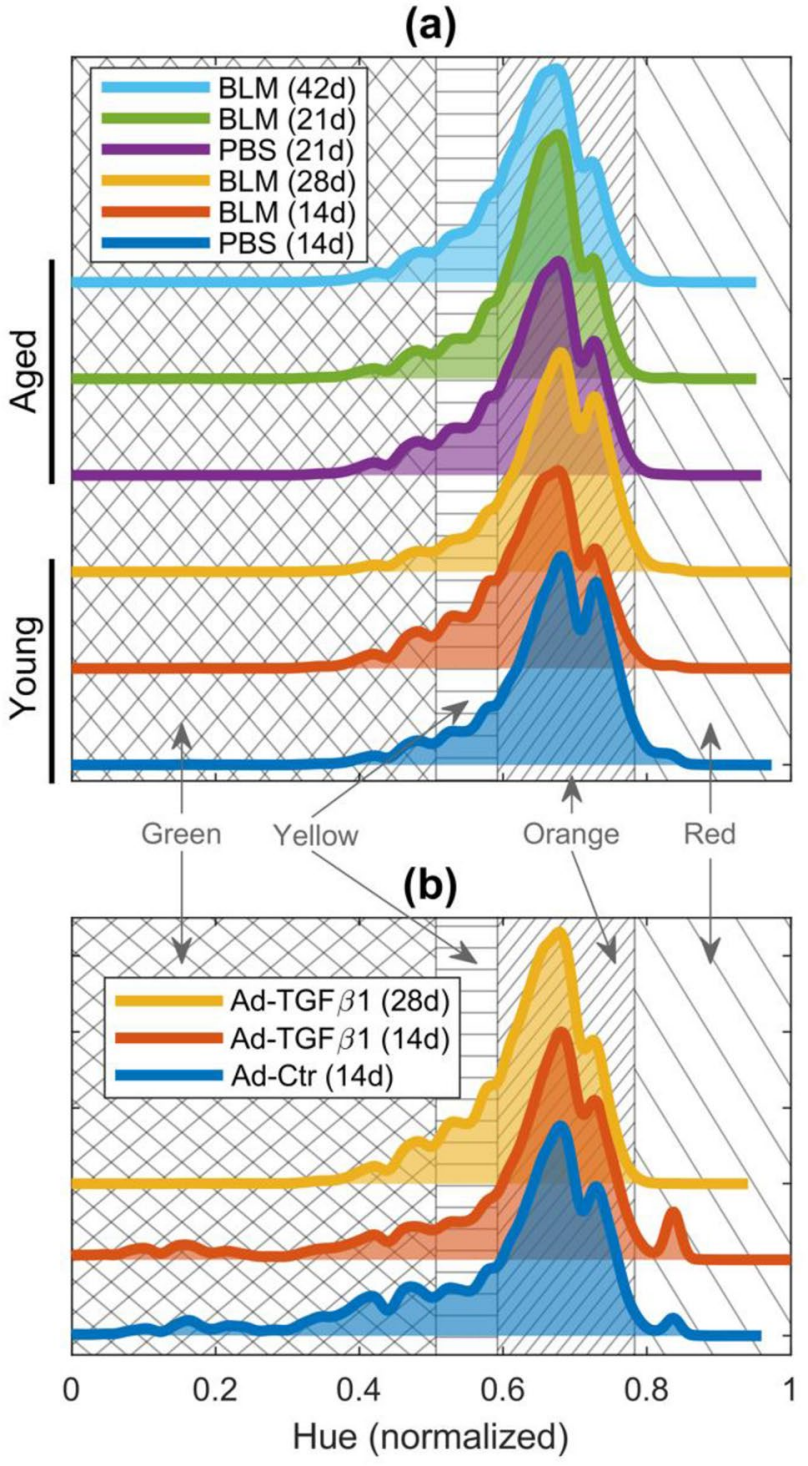
Normalized hue distribution of polarized PSR for **a** bleomycin-induced experiment of young and aged mice, **b** TGFβ1-induced experiment. All distributions have an area of 1 and are offset vertically for visualization. The background hatching marks the normalized hue values that fall into distinct categories for collagen thickness. In increasing order of collagen thickness: cross hatching is for green hues, horizontal hatching is for yellow hues, tight right-diagonal hatching is for orange hues, and wide left-diagonal hatching is for red hues

**Fig. 6:**
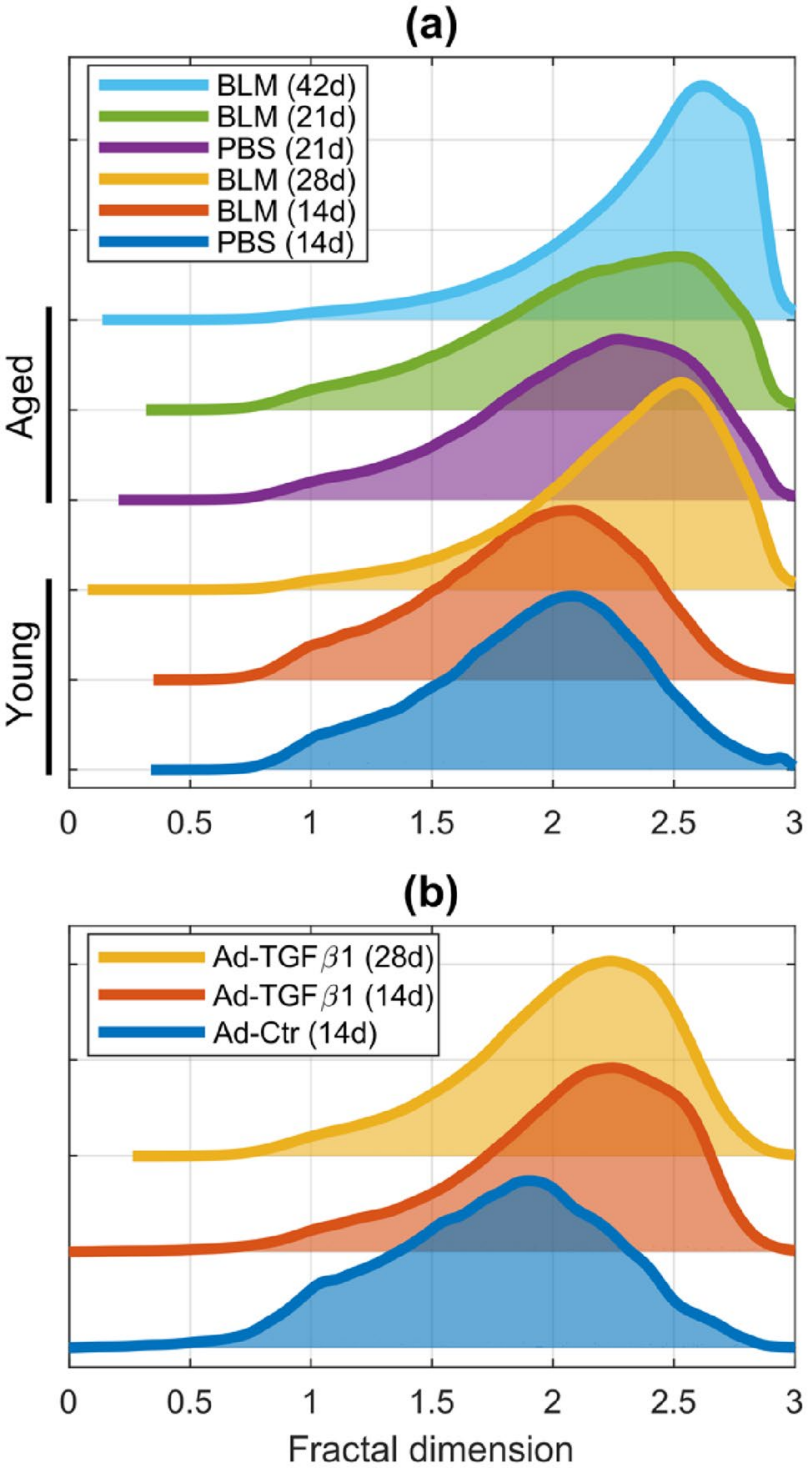
LCFSD distributions for **a** bleomycin-induced experiment of young and aged mice, **b** TGFβ1-induced experiment. All distributions have an area of 1 and are offset vertically for visualization

The distributions in Figs. 5 and 6 aggregate all images from all animals within a specific group. Kernel density plots of normalized hue values are shown in Fig. 5. Within each experiment, the hue distributions for each group showed no significant differences (Table 1, top two rows). Kernel density plots of the fractal dimension distributions are shown in Fig. 6. Most of the distributions are left-skewed but are right-shifted in the aged and fibrotic groups, indicative of higher fractal dimensions (Fig. 6). Differences in the LCFSD distributions for each experiment are summarized in the bottom two rows in Table 1. Three pairs summarized in Fig. 6a, young PBS with young BLM 14d, aged PBS 21d with aged BLM 21d, and young BLM 28d with aged BLM 42d tested significantly different from other groups, but not from each other. All other pairwise comparisons were significantly different with* p* < 0.025. The only significant differences in the Ad-TGFβ1 experiment (Fig. 6b) were between the control group, Ad-Ctr, with the groups Ad-TGFβ1 14d (*p* = 0.0008) and Ad-TGFβ1 28d (*p* = 0.0363).

**Table 1:** Kruskal–Wallis test results for hue distributions from Fig. 5 in top two rows. Kruskal–Wallis test results for fractal dimension distributions from Fig. 6 in bottom two rows

	Experiment	*N*	Chi-squared	Degrees of freedom (df)	*p* value
Hue distributions	Bleomycin-induced	100	7.09	5	0.2141
	TGFβ1-induced	100	0.45	2	0.7994
Fractal distributions	Bleomycin-induced	100	118.45	5	< 0.0001
	TGFβ1-induced	100	13.82	2	0.0010

**Fig. 7:**
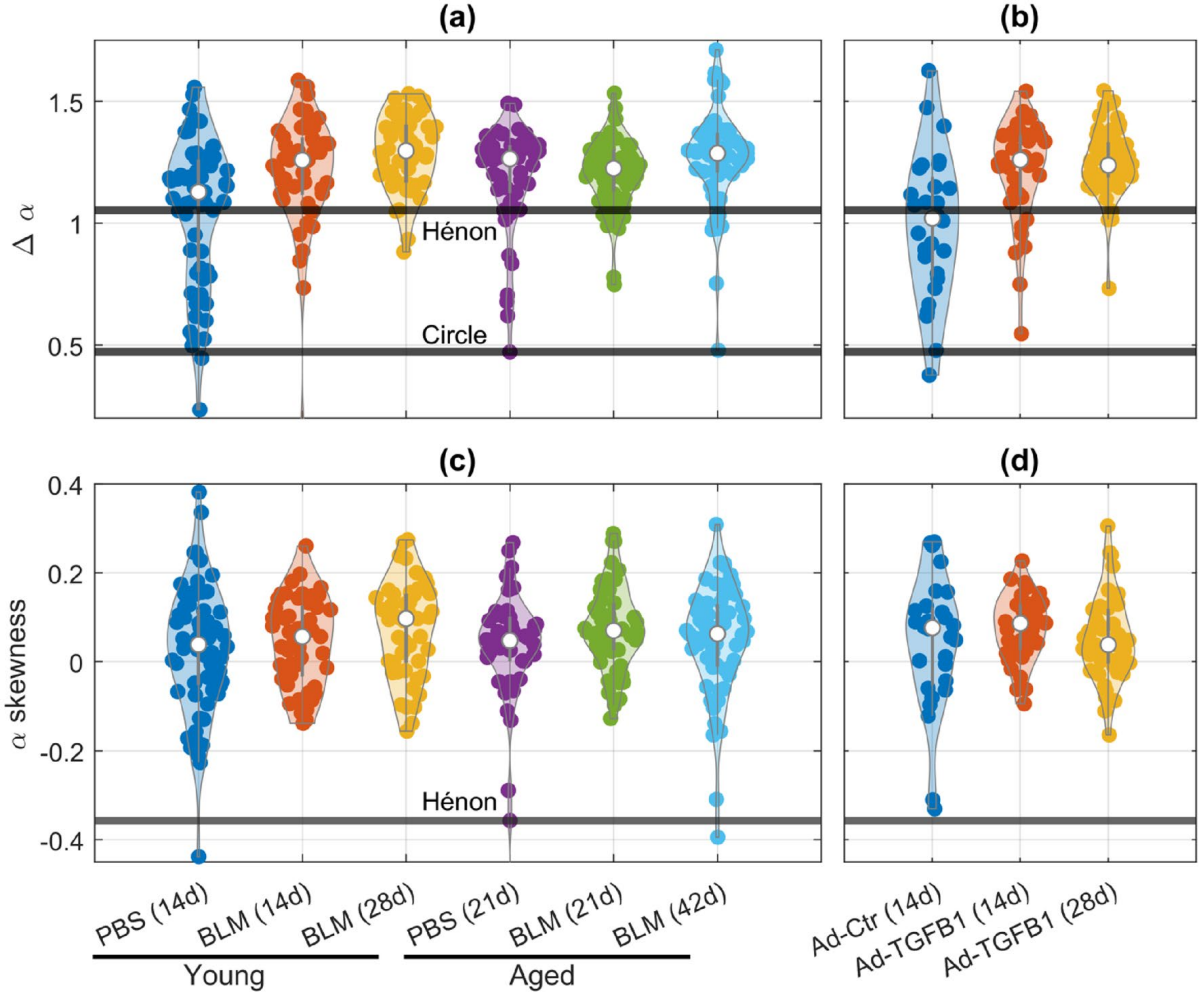
Δα for each image, with Hénon map and circle references for **a** bleomycin-induced experiment of young PBS 14d (*n* = 70), BLM 14d (*n* = 50), and BLM 28d (*n* = 50) mice and aged PBS 21d (*n* = 50), BLM 21d (*n* = 60), and BLM 42d (*n* = 60) mice; **b** TGFβ1-induced experiment with groups Ad-Ctr 14d (*n* = 30), Ad-TGFβ1 14d (*n* = 40), and Ad-TGFβ1 42d (*n* = 50). α-skewness for each image using Eq. (5), with Hénon map reference for **c** bleomycin-induced experiment of young PBS 14d (*n* = 70), BLM 14d (*n* = 50), and BLM 28d (*n* = 50) mice and aged PBS 21d (*n* = 50), BLM 21d (*n* = 60), and BLM 42d (*n* = 60) mice; **d** TGFβ1-induced experiment with groups Ad-Ctr 14d (*n* = 30), Ad-TGFβ1 14d (*n* = 40), and Ad-TGFβ1 42d (*n* = 50)

All images in each group had normally distributed $\Delta \alpha$ values (Fig. 7a, b). Besides the Ad-Ctr group (Fig. 7b), the median value of $\Delta \alpha$ for each group was higher than the corresponding Hénon map, signifying that most images were multifractal in nature. Some images appear near the $\Delta \alpha$ for a circle, meaning they were similar to mono-fractals. The $\alpha$-skewness for all groups was normally distributed with a median near zero (Fig. 7c, d).

## Discussion

Multifractal analysis demonstrates multiple scales of fractal structure in images, but it has the drawback of being a global metric that cannot quantify heterogeneity within an image. The LCFD would be more appropriate for capturing spatial variations in fractal structure if it were not for the fact that it can only be applied to binary images. This motivated us to extend the LCFD into the third dimension to allow it to account for collagen thickness. Accordingly, we designed a new metric of fractal dimension, the LCFSD, in order to analyze planar images that contain meaningful information encoded in their pixel intensities. We validated the LCFSD by applying it to heterogeneous structures known as multifractals with varying local spatial complexity. We then applied the LCFSD to histologic images of fibrotic lung tissue and found that fibrotic regions of tissue have higher fractal dimensions compared to non-fibrotic regions, indicating that the tissue remodeling in the fibrotic regions is associated with increased spatial complexity (Fig. 3). We also found that modifications of collagen structure can be detected independent of collagen content.

We chose to demonstrate the LCFSD using Julia sets because of their unique formation. These fractals are defined on the complex plane with their iterative formation as defined by Eq. (4) determining whether a point in the complex plane is a member of the set (Fatou 1917; Julia 1918; Brolin 1965). However, each point in a Julia set has a bounded value representing how close the value is from being unbounded and being excluded from the set. We were able to use the value of each point as a third dimension for a planar image, which our LCFSD was developed to analyze. We selected parameters of the Julia sets to display certain properties such as connectedness (Fig. 1), nonfractal third dimension (Fig. 2a), and fractal third dimension (Fig. 2b). Each parameter was iterated until the Julia set was clearly defined in our 1386 × 1386-pixel complex plane. In the case of the Julia set defined by $c=-1.15+0.215i$, the pixel values would shift between two extremes during its orbit, giving this planar fractal two distinct fractal surfaces. We report the results of the next iteration in Supplementary Fig. 2, which shows the same behavior as Fig. 2d, f. Furthermore, the change to the bimodal distribution solely from a change in the pixel values demonstrates the sensitivity of the LCFSD.

It is important to note that the hydroxyproline assay and PSR quantification do not assess the same areas of the lung. When the whole lung is homogenized, it includes large amounts of collagen from airways and blood vessels in addition to that derived from the parenchyma. When bleomycin is administrated intratracheally it causes an increase in collagen content around the bronchi, as is evident from PSR imaging (Borzone et al. 2001). Fibrosis of airways, however, is not a hallmark feature of IPF (Martinez et al. 2017). Histological quantification deliberately avoids these areas and focuses exclusively on the parenchyma. Also, the hydroxyproline assay and PSR quantification do not detect collagen in the same manner despite both increasing linearly with collagen content (Neuman and Logan 1950; Junqueira et al. 1979b). The hydroxyproline assay measures the amount of the amino acid hydroxyproline, which is mostly found in collagen but does appear sparsely in other proteins (Neuman and Logan 1950). In contrast, Sirius Red is an elongated molecule with six sulfonic groups that specifically aligns parallel to collagen molecules by interacting with their basic groups, causing them to become birefringent (Junqueira et al. 1979a). Shorter wavelengths are associated with more collagen whether it be from thicker or denser fibers (Junqueira et al. 1979b). Therefore, the results of LCFSD analysis are likely affected by the choice of stain used to visualize collagen in a histologic image.

A Picrosirius red stain of any connective tissue is exclusive to collagen when viewed under circularly polarized light. The added benefit of this technique is that the pixel intensity value is also a rough measure of fiber thickness (Rich and Wittaker 2005). It has previously been used to quantify collagen content as the summation of the pixels from histopathology of a fibrotic lung (Anathy et al. 2018). While fibrosis is commonly thought of as an excess of collagen deposition, it is now recognized that aberrant crosslinking and reorganization of existing collagen are factors that contribute to disease progression (Jones et al. 2018; Casey et al. 2021). Hence, quantifying changes in tissue structure via a fractal dimension may provide an alternative metric of phenotypic changes in fibrosis to complement the conventional metric of total collagen. Furthermore, the histology of pulmonary fibrosis, particularly IPF, is characterized by spatial heterogeneity in which healthy tissue is often adjacent to fibrotic regions (Raghu et al. 2018). This information is lost in global fractal metrics that aggregate disparate structures into a single measure, which can be overcome by using LCFSD to quantify local variations in collagen organization and thickness in PSR-stained tissue.

The similarity between the hue distributions in Fig. 5 shows that these distributions are not only independent of age and condition but also of how much collagen is present in the tissue. These characteristics may be a consequence of how collagen fibers develop, although it is not clear whether this is unique to the mouse lung. Lung tissue is composed mostly of type I and type III collagens (Laurent 1986). Both types produce strong fibers, but there is no particular correlation between hue and collagen type (Junqueira et al. 1978). The vast majority of hue values fall in the orange category followed by, in decreasing order, yellow, green, and red. It is also difficult to know if there is any significance associated with the finer details in these distributions, such as the second smaller peak that appears to the right of the main peak or the waves that occur in the yellow and green hue regions (Fig. 5). Although a similar behavior has also been seen in heart tissue, suggesting that it may be a feature of tissue collagen in general, it can also be artifactual due to the 8-bit binning of intensity values (MacKenna et al. 1994).

To examine how collagen remodeling progresses with time, we examined tissue from two time points for each experiment, which was verified by hydroxyproline content increases with induction of fibrosis relative to a control at 14d or 21d time point, and further increases at the 28d or 42d time point. The different methods that were used to induce fibrosis resulted in different changes in the nature of lung tissue collagen. We find that parenchymal changes analyzed via histology (Figs. 4, 5, 6) did not provide an accurate surrogate for the total collagen content of the lung quantified through hydroxyproline content (Fig. 4a, b). Instead, they provided additional evidence of the structural transformations of collagen.

The earlier time points (14d/21d) in the bleomycin-induced experiment showed increased hydroxyproline content compared to control in both young and aged mice (Fig. 4a), yet the collagen percent areas were the same in the four groups (Fig. 4c). The substantially elevated collagen percent areas in the late time point groups (28d/42d) suggest that increased parenchymal collagen deposition occurs in the later stages of fibrosis. The aged mouse groups generally had much intra-animal variability in mean collagen percent area (Fig. 4c) reflected in large within-animal standard deviations (Fig. 4e). This is reminiscent of the diversity and heterogeneity seen between patients with IPF (Ley et al. 2011).

The fractal dimension distributions (Fig. 6a) followed trends similar to those of the PSR quantification while at the same time providing structural information. The young BLM 28d fractal distribution was skewed more heavily to the left than the young PBS 14d and BLM 14d distributions. Those two 14d groups had statistically indistinguishable distributions centered near a value of 2. The trend was the same between the aged groups where the distributions for PBS 21d and BLM 21d were identical while the aged BLM 42d distribution was skewed to higher fractal dimensions. Both cases demonstrated an increase in tissue complexity with the progression of fibrosis. Furthermore, the fractal distributions of the aged 21d mice were initially more skewed than their young 14d counterparts, suggesting that aging itself contributes to the complexity of the collagen structure. Why this happens is unclear, but presumably it somehow reflects the accumulated imperfections of ongoing tissue maintenance throughout life (Onursal et al. 2021).

The changes in collagen content in the Ad-TGFβ1-induced experiment (Fig. 4b) were not seen in the collagen percent area quantification in the same animals (Fig. 4d). Means and distributions of collagen percent area were consistent between animals and conditions, with minimal deviations from the intra-animal and inter-animal means (Fig. 4f). However, the fractal analysis of the same images (Fig. 6b) produced distributions that mimic the changes in the biochemical quantifications (Fig. 4b). Both the Ad-TGFβ1 14d and 28d groups had similar fractal dimension distributions that skewed toward higher fractal dimensions relative to the control (Fig. 6b). When considered together with the hydroxyproline data, these trends suggest that the induction of fibrosis in this model occurred acutely but was also long lasting. The fractal dimension distributions in the Ad-TGFβ1-induced experiment did not have values as high as in the bleomycin-induced experiment (Fig. 6a), indicating that the Ad-TGFβ1 model of fibrosis might have less complex collagen structure. Higher fractal dimensions in the Ad-TGFβ1-induced experiment may thus be reflective of remodeling caused by the overproduction of TGFβ1 via the adenovirus and not the increase in parenchymal collagen content measured via histology. Hence, the LCFSD reveals the presence of collagen reorganization independent of both amount of collagen and thickness of the fibers.

Our new LCFSD method has some limitations and caveats. It does not provide useful insight in non-fractal third-dimensional situations such as pertains to the left-hand side of Fig. 2. The metric is independent of the number of pixels in an image or set of images, so other methods should be used for quantification of collagen content. LCFSD must also be applied judiciously to situations where fibrotic disease is spatially heterogeneous, as is the case of IPF. Care must also be taken to avoid irrelevant structures such as airways and blood vessels, highlighting the importance of the processing procedure prior to applying the LCFSD method. This can introduce some degree of subjectivity into the selection of regions of interest.

Another way subjectivity can be introduced is with selecting $h$, the max box size for the calculation. This choice may be influenced by the length scale of the phenomena of interest. For instance, we could use a higher $h$ to capture collagen fibers longer than 6.3 μm. A more objective way to select $h$ is to use the cluster distribution sizes to determine a side length that incorporates most structures. In our case, the BLM 28d/42d groups were skewed higher because they contained larger clusters of collagen (Supplementary Fig. 1). Choosing a higher $h$ value might obscure smaller features because $N(L)$ would not change for small clusters at higher side lengths. Choosing an $h$ too small would make distinguishing features more challenging because more intricate structures would be excluded. The scale of the image is also important to consider because if we took our images at ×40, our side length would have to be about double to capture the same area, albeit at higher resolution.

The distributions of fractal dimension produced by LCFSD are also dependent to some degree on the technique used to highlight structures of interest. This applies to the PSR versus hydroxyproline staining of the present study, but LCFSD is also applicable to other imaging modalities that produce quantitative pixel values such as CT. This means that comparison of LCFSD results between different treatments is only likely to be meaningful if all other experimental conditions are the same.

We have developed a novel method, the LCFSD, for calculating the local fractal dimension in histopathological images that exploits the information inherent in pixel intensities to reveal regions of local complexity in multifractal objects. When applied to images of fibrotic lung tissue, the LCFSD shows increases in the fractal dimensions of local collagen structures despite collagen fiber thickness remaining unchanged. Tissue remodeling is thus clearly not simply a matter of altered collagen content; alterations in its structure and topographic arrangement may be just as important and can thus serve as important indicators of remodeled tissue. The LCFSD has the potential to be a useful marker of these changes.

### Supplementary Information

Below is the link to the electronic supplementary material.

Supplementary file1 (DOCX 211 KB)

## Data Availability

The dataset analyzed during the current study is available from the corresponding author on reasonable request. Code used to perform this analysis is available at https://github.com/dylantcasey/LCFSD.
