# Supplementary material for: Local fractal dimension of collagen detects increased spatial complexity in fibrosis
Source: Histochem Cell Biol. 2023 Nov 8;161(1):29–42. doi: 10.1007/s00418-023-02248-8 (PMC10794291; doi:10.1007/s00418-023-02248-8)
Supplement: Supplementary file 1 — Supplementary file1 (DOCX 211 KB) [file 418_2023_2248_MOESM1_ESM.docx]

Supplemental Data


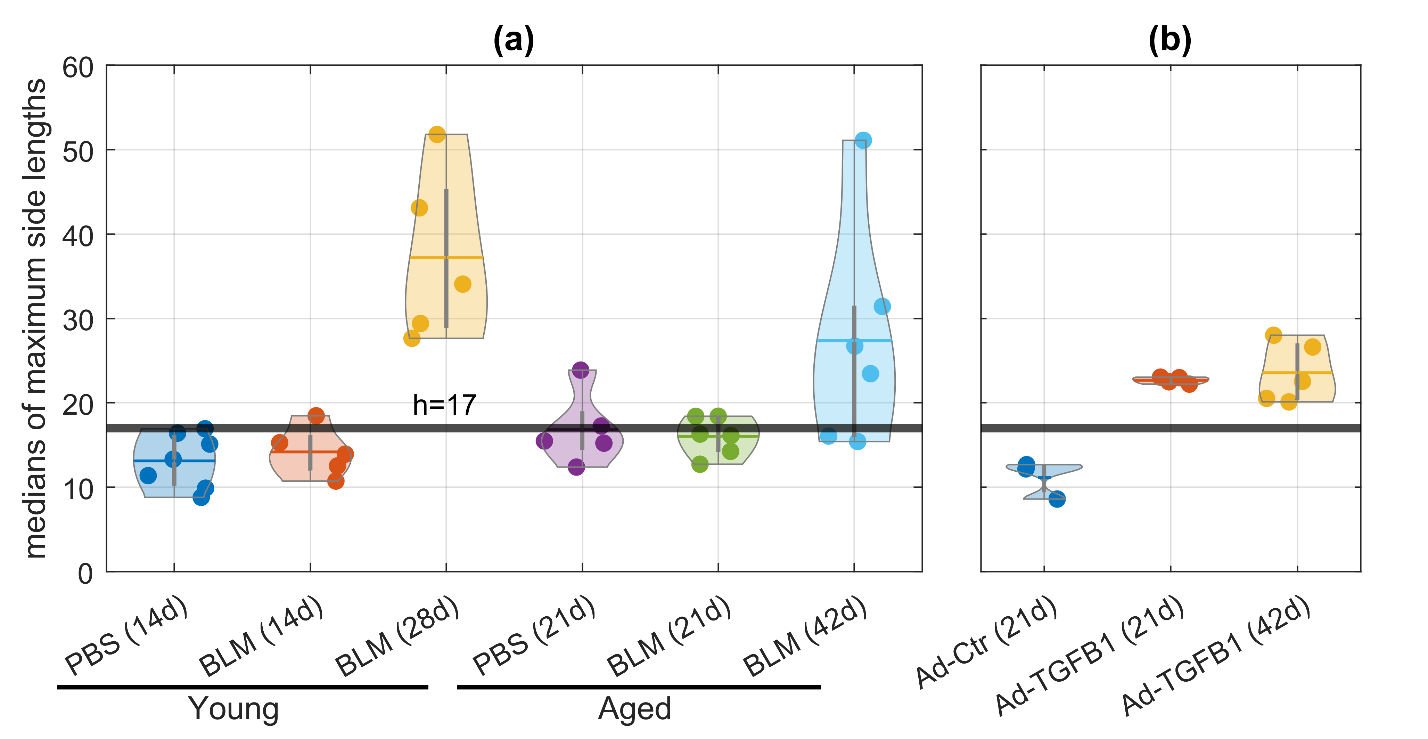


Sup Fig 1 Distribution of the square root of the largest pixel cluster for each image. Each point represents the median of individual images. Black horizontal line reflects the chosen side length of $\boldsymbol{h=17}$ for LCFSD calculations. Medians of maximum side lengths for (a) bleomycin-induced experiment of young PBS 14d (n=7), BLM 14d (n=5), and BLM 28d (n=5) mice and aged PBS 21d (n=5), BLM 21d (n=6), and BLM 42d (n=6) mice and (b) TGFβ1-induced experiment with groups Ad-Ctr 21d (n=3), Ad-TGFβ1 21d (n=4), and Ad-TGFβ1 42d (n=5) from Anathy et al. (2018)


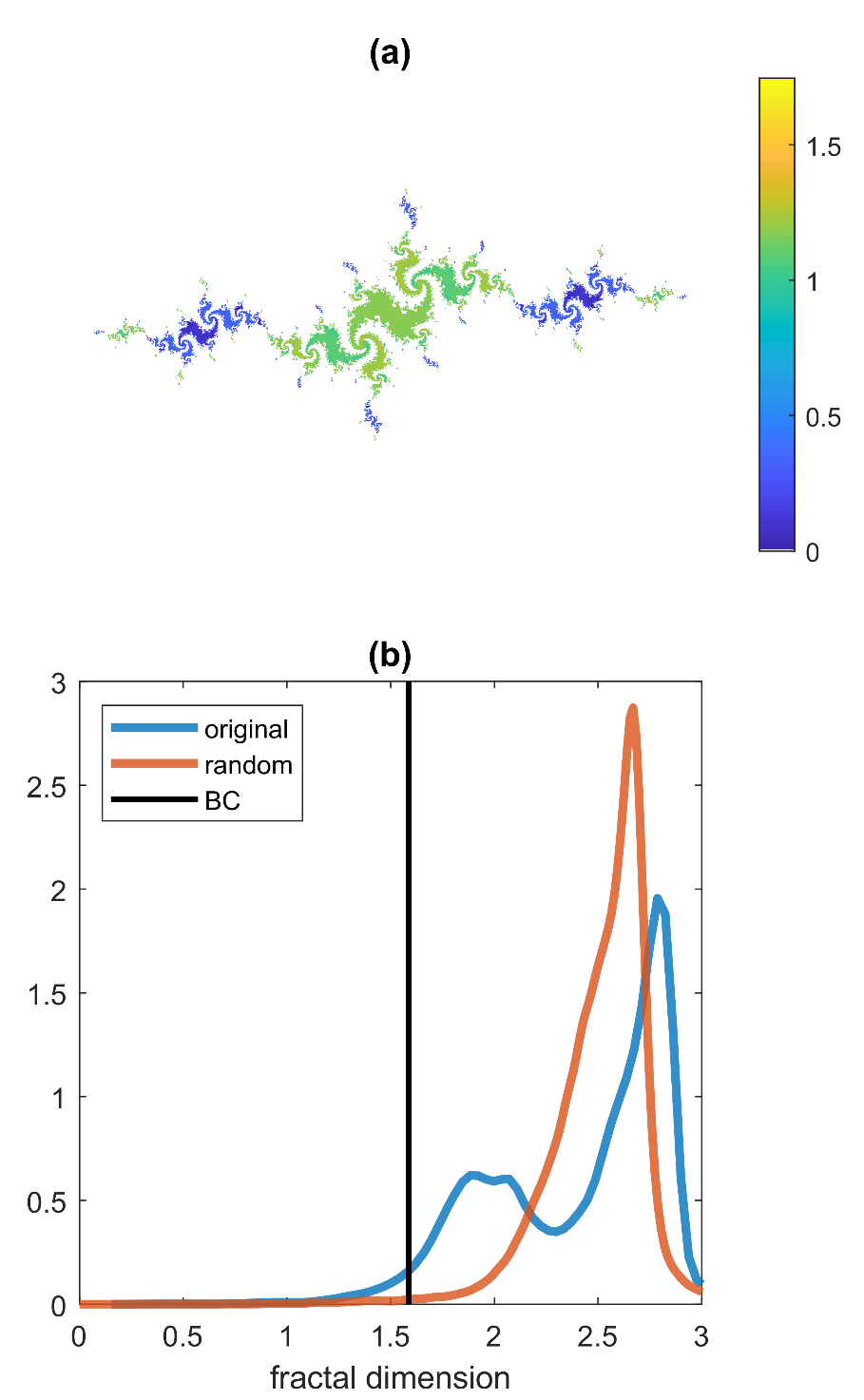


Sup Fig 2 LCFSD applied to Julia set for $\boldsymbol{c=-1.15+0.215}\boldsymbol{i}$ at iteration 97 (a) colored by magnitudes. Coloration by escape iteration shown in Fig 2b. (b) LCFSD distribution for Julia set in a, including box-counting dimension in black (BC) and LCFSD of randomized magnitudes in orange
